# The Impact of Halogen Substituents on the Synthesis and Structure of Co-Crystals of Pyridine Amides

**DOI:** 10.3390/molecules26041147

**Published:** 2021-02-21

**Authors:** Amila M. Abeysekera, Abhijeet S. Sinha, Christer B. Aakeroy

**Affiliations:** Department of Chemistry, Kansas State University, Manhattan, KS 66506, USA; abeysekeramila@ksu.edu (A.M.A.); sinha@ksu.edu (A.S.S.)

**Keywords:** hydrogen bonds, halogen bonds, crystal engineering, molecular recognition

## Abstract

Strategies for co-crystal synthesis tend to employ either hydrogen- or halogen-bonds between different molecules. However, when both interactions are present, the structural influence that they may exert on the resulting assembly is difficult to predict a priori. To shed some light on this supramolecular challenge, we attempted to co-crystallize ten aliphatic dicarboxylic acids (co-formers) with three groups of target molecules; *N*-(pyridin-2-yl)picolinamides (2Pyr-*X*), *N*-(pyridin-2-yl)nicotinamides (3Pyr-*X*), *N*-(pyridin-2-yl)isonicotinamides (4Pyr-*X*); X=Cl/ Br/ I. The structural outcomes were compared with co-crystals prepared from the non-halogenated targets. As expected, none of the reactions with 2Pyr-*X* produced co-crystals due to the presence of a very stable intramolecular N-H···N hydrogen bond. In the 3Pyr series, all six structures obtained showed the same synthons, –COOH···N(py) and –COOH···N(py)-NH, that were found in the non-halogenated parent 3Pyr and were additionally accompanied by structure directing X···O(OH) interactions (X=Br/I). The co-crystals of the unhalogenated parent 4Pyr co-crystals assembled via intermolecular –COOH···N(py) and –COOH···N(py)-NH synthons. Three of the analogues 4Pyr-X co-crystals displayed only COOH···N(py) and –COOH···N(py)-NH interactions. The three co-crystals of 4Pyr-X with fumaric acid (for which no analogues structures with 4Pyr are known) formed –COOH···N(py)-NH and –NH···O=C hydrogen bonds and showed no structure-directing halogen bonds. In three co-crystals of 4Pyr-I in which –COOH···N(py)-NH hydrogen bond was present, a halogen-bond based –I···N(py) synthon replaced the –COOH···N(py) motif observed in the parent structures. The structural influence of the halogen atoms increased in the order of Cl < Br < I, as the size of σ-holes increased. Finally, it is noteworthy that isostructurality among structures of the homomeric targets was not translated to structural similarities between their respective co-crystals.

## 1. Introduction

Practical crystal engineering is driven by the use of intermolecular forces, primarily hydrogen-bonding [1], for connecting discrete molecular building blocks or coordination complexes into extended crystalline architectures [2,3,4,5]. A “second-phase” of research in this area has added (i) synthetic complexity by taking advantage of additional structure-directing interactions such as halogen [6] and chalcogen bonds [7] and (ii) compositional complexity by building multi-component architectures such as binary, ternary [8,9,10], and higher-order co-crystals [11]. Most of these efforts have been in the area of basic science, with the goal of establishing guidelines for how different molecules and functional groups recognize and bind to each other via non-covalent interactions [12]. Such insights can enable forays into applied materials science by applying co-crystal technology to the synthesis of new solid forms of pharmaceutically relevant compounds [13,14], agrochemicals [15,16], energetic materials [17,18], and other high-value solids [19]. In order to learn more about competing molecular-recognition events in supramolecular assembly, we previously examined the outcomes of co-crystallization of *N*-(pyridin-2-yl)nicotinamide and *N*-(pyridin-2-yl)isonicotinamide with di-carboxylic acids and identified reliable recognition events between both the N_2_(py) and N_1_(py)—NH sites of the target molecules to the dicarboxylic acids. Additionally, the fundamental nature of the observed architectures (rings or chains) could be correlated to the geometries of the binding sites of target molecules and the relative position of —COOH groups in odd and even chained acids [20].

**Figure 1:**
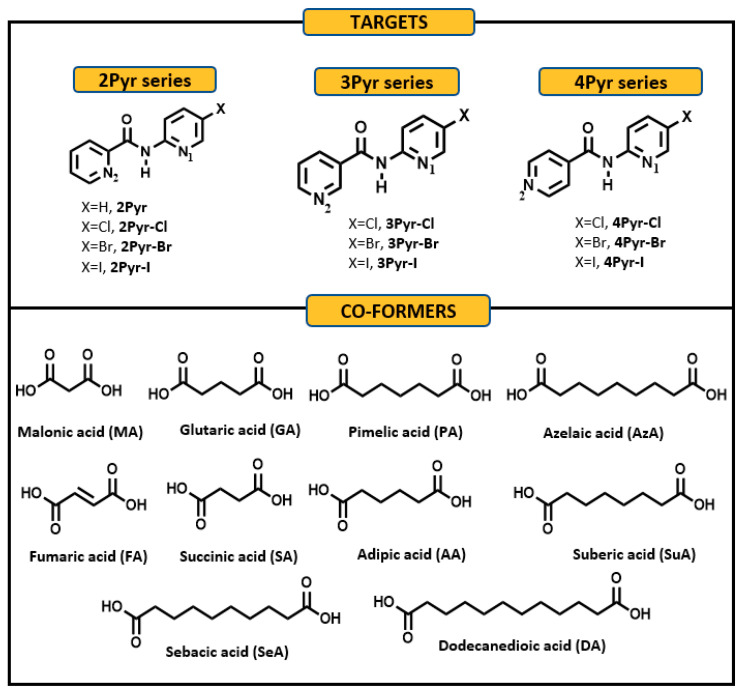
Schematics of target molecules (top) and co-formers (bottom) in this study.

In order to expand and refine practical crystal engineering strategies, we wanted to systematically map out the structural influence exerted by a potential halogen bonding site on the supramolecular framework in the same set of target compounds. Furthermore, we also wanted to establish if it is reasonable to hypothesize that differences between crystal structures of halogenated and non-halogenated homomeric assemblies would lead to similar differences in co-crystals of the same set of targets. The work presented herein utilized a systematic structural study of co-crystals of closely related compounds, Figure 1, in order to learn more about the balance between competing interactions in heteromeric molecular architectures. 

**Figure 2:**
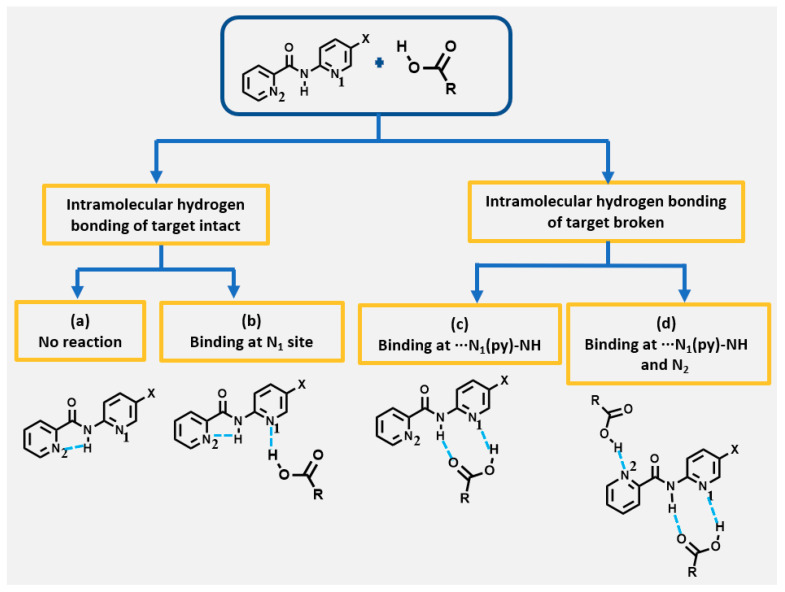
Possible outcomes of co-crystallization experiments of 2Pyr series compounds. Outcomes (**a**) No reaction and (**b**) Binding at N_1_ may occur with intramolecular hydrogen bonding intact. Outcomes (**c**) Binding at ···N_1_(py)-NH and (**d**) Binding at ···N_1_(py)-NH and N_2_(py) are assumed not to take place because of the likely presence of an intramolecular hydrogen-bonded five-membered ring.

We previously identified the prevailing hydrogen-bond interactions in the ten targets [21]. The 2Pyr compounds all displayed intramolecular N-H···N_2_ hydrogen bonding, whereas this was not geometrically possible in the 3Pyr/4Pyr analogues. This allowed us to hypothesize that even though all three isomers contain identical hydrogen-bonding sites, the presence of an intramolecular five-membered “ring” in 2Pyr would preclude these target compounds from forming co-crystals with carboxylic acids through pairs of N-H···O=C/N···H-O hydrogen bonds (Figure 2). 

In this study we wanted to address five specific questions.

⮚ What is the effect of co-crystallization ability in targets where the NH hydrogen of the N_1_(py)-NH pocket is engaged in intramolecular hydrogen bonding to N_2_ (Figure 2)?
⮚ How does the presence of halogen atoms alter the supramolecular assembly of co-crystals?
⮚ Are any observed alterations consistent amongst targets in the same series?
⮚ Are there instances in which the (potential) halogen-bond donor competes with the hydrogen-bond donor?
⮚ Do isostructural targets exhibit isostructurality in their corresponding co-crystals?

**Figure 3:**
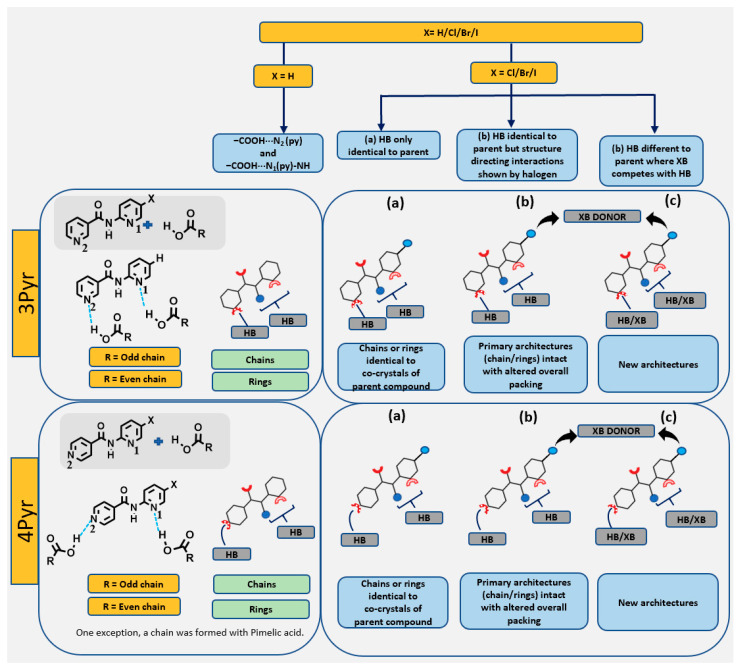
Postulated structural outcomes in co-crystals of halogenated target molecules in 3Pyr/4Pyr series and carboxylic acids. HB = hydrogen bonding, XB = halogen bonding where (**a**) HB only, (**b**) HB and structure directing non-competitive XB interactions, (**c**) XB competes with HB leading to new architecture with halogenated 3Pyr (top) and 4Pyr (bottom) targets respectively.

Three possible outcomes in the presence of a halogen atom on the molecular framework of co-crystals in 3Pyr and 4Pyr series may be postulated, as shown in Figure 3.

## 2. Results

The outcome of the IR screening results is given in Table 1. The relevant IR data is provided in the Supplementary Materials.

The resultant solids that showed co-crystal formation were dissolved in a variety of solvents to obtain crystals of diffraction quality; see Table 2 for details. Out of the 47 co-crystals that were obtained, 15 produced crystals suitable for single-crystal X-ray diffraction (SCXRD). Despite much effort, only two co-crystals with odd-chain carboxylic acids were obtained. The difficulty of growing crystals with odd-chain carboxylic acids has been previously noted [22,23].

**Figure 4:**
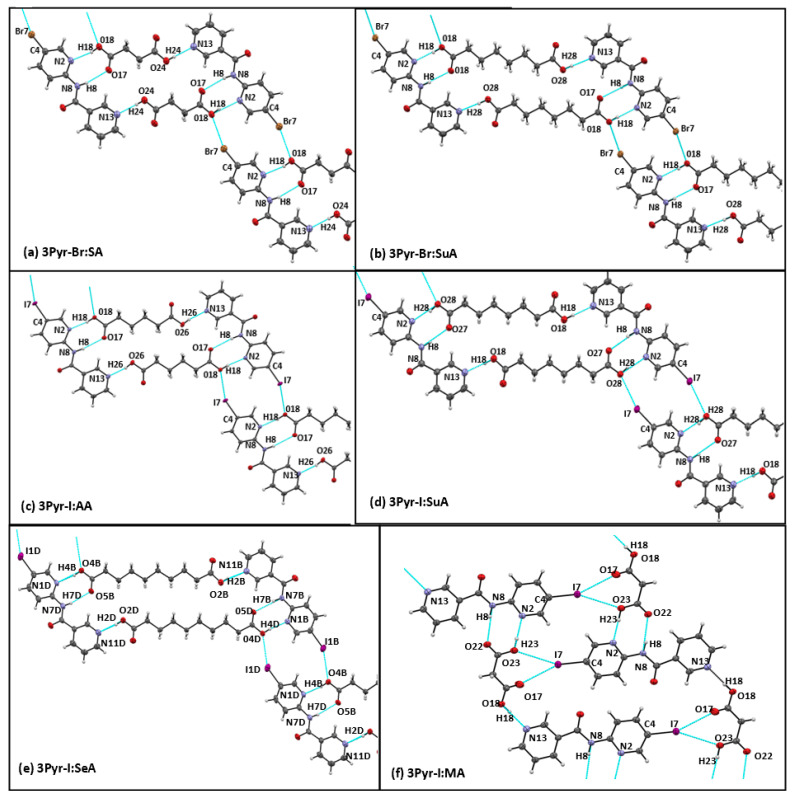
Primary interactions seen in single crystal structures of (**a**) 3Pyr-Br:SA, (**b**) 3Pyr-Br:SuA, (**c**) 3Pyr-I:AA, (**d**) 3Pyr-I:SuA, (**e**) 3Pyr-I:SeA*, and (**f**) 3Pyr-I:MA. * Please see Supplementary Materials for image with all crystallographically unique molecules displayed.

Six co-crystals of 3Pyr-X targets suitable for SCXRD were successfully grown. In the five co-crystals with even-chain acids (3Pyr-Br:SA, 3Pyr-Br:SuA, 3Pyr-I:AA, 3Pyr-I:SuA, 3Pyr-I:SeA) and the one with an odd-chain acid (3Pyr-I:MA), each target interacted with two acid molecules. With the even chain acids, 3Pyr-Br and 3Pyr-I formed two identical synthons, –COOH···N_2_(py) and –COOH···N_1_(py)-NH, which overall led to a tetrameric ring (Figure 4a–e) in a 1:1 stoichiometric ratio. In addition, both Br and I engaged in halogen bonds to the hydroxylic oxygen, thereby linking neighboring rings into an infinite ribbon (Figure 3a–e). The Br···O interactions showed a reduction of combined van der Waals radii of 5%, while the I···O showed a 10% reduction. In the structure of 3Pyr-I:MA, the hydrogen bonds (HBs) were once again –COOH···N_2_(py) and –COOH···N_1_(py)-NH but now led to chain-like architectures. The iodine atom formed two halogen bonds to both OH groups of a neighboring carboxylic acid molecule with van der Waals reductions of 10% and 3%, respectively. Thus, the halogen bonding effectively linked chains together (Figure 4f).

The relevant hydrogen- and halogen-bond geometries in the six co-crystals from the 3Pyr series are given in Table 3.

**Figure 5:**
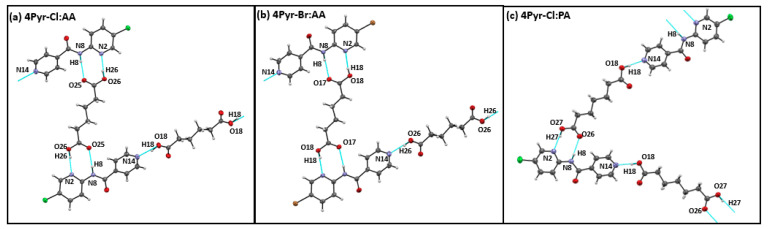
Primary interactions seen in single crystal structures of (**a**) 4Pyr-Br: AA, (**b**) 4Pyr-Br: AA, (**c**) 4Pyr-Cl-PA.

A total of nine co-crystals of 4Pyr-X targets suitable for SCXRD were successfully grown. Eight of these contained even-chain acids as co-formers. Except for three of the iodinated co-crystals (4Pyr-I:AA, 4Pyr-I:SuA, and 4Pyr-I:SeA), each target molecule in all the other co-crystals formed hydrogen bonding to two carboxylic acid molecules. In the co-crystals of 4Pyr-Cl:AA, 4Pyr-Br:AA, and 4Pyr-Cl:PA, two identical hydrogen-bonding (HB) interactions, −COOH···N_2_(py) and −COOH···N_1_(py)-NH, with the carboxylic acid led to chain-like architectures. There were no structure directing interactions shown by bromine or the chlorine atoms in these structures, Figure 5a–c.

**Figure 6:**
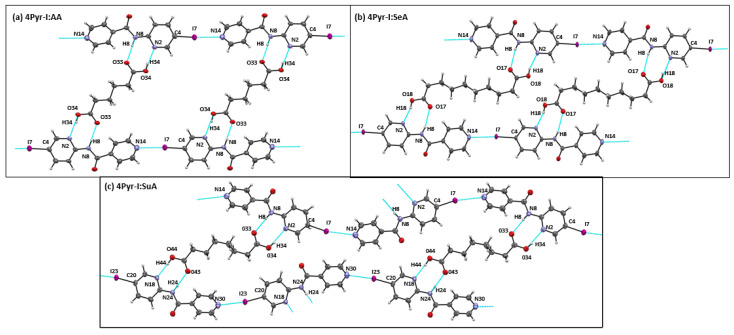
Primary interactions seen in single crystal structures of (**a**) 4Pyr-I:AA*, (**b**) 4Pyr-I:SeA, (**c**) 4Pyr-I:SuA. * Please see Supplementary Materials for image with all crystallographically unique molecules displayed.

In three co-crystals 4Pyr-I:AA, 4Pyr-I:SuA, and 4Pyr-I:SeA, only −COOH···N_1_(py)-NH interaction was seen between the target and co-former. In addition, −I···N_2_ (py) intermolecular interactions were formed between target molecules. With each acid, the target compound combined in a 2:1 stoichiometry. The overall architecture can be described as having a “ladder-like” architecture (Figure 6a–c). Within these three co-crystals, 4Pyr-I:SuA contrasted to the other two as it had the carbonyl groups of halogen-bonded target molecules arranged trans to each other.

**Figure 7:**
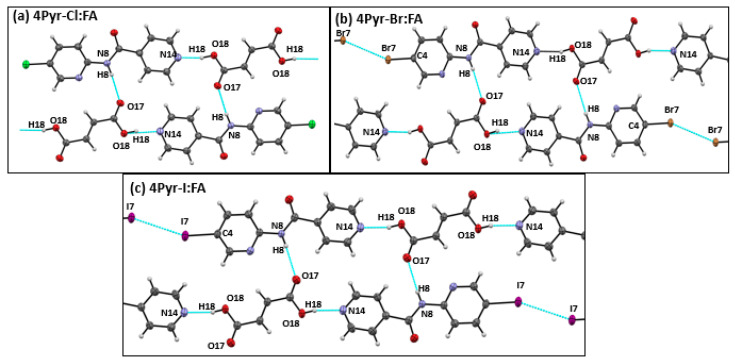
Primary interactions in crystal structures of (**a**) 4Pyr-Cl:FA, (**b**) 4Pyr-Br:FA, and (**c**) 4Pyr-I:FA.

The three co-crystals of 4Pyr-Cl:FA, 4Pyr-Br:FA, and 4Pyr-I:FA showed identical HB patterns. Both the N_1_(py)-NH and N_2_(py) sites were engaged in hydrogen bonding to the carboxylic acid. Each target bound to two carboxylic acid molecules, forming −COOH···N_2_(py) and −NH···O=C hydrogen bonds (Figure 7). In addition, a halogen–halogen (Type I) interaction was seen in both 4Pyr-Br:FA and 4Pyr-I:FA, with van der Waals reductions of 2% and 8%, respectively.

The relevant hydrogen- and halogen-bond geometries in the nine co-crystals from the 4Pyr series are given in Table 4.

## 3. Discussion

**Figure 8:**
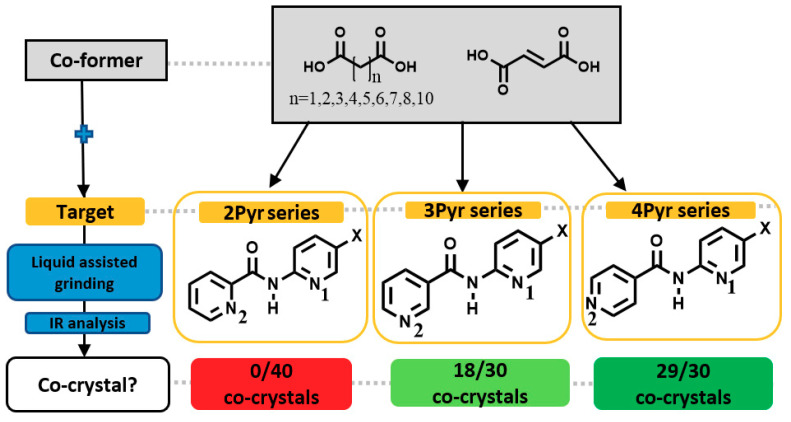
Summarized results of co-crystal screen.

None of the compounds in the 2Pyr series formed co-crystals while eighteen out of thirty experiments gave positive results in the 3Pyr series and twenty-nine out of thirty experiments gave positive results for the 4Pyr series halogenated compounds (Figure 8).

The absence of co-crystal formation by any compound in the 2Pyr series demonstrates the stability of this five-membered hydrogen-bonded N-H···N_2_ ring, which effectively precludes intermolecular hydrogen bonding (essential for co-crystal synthesis) at that binding site. In addition, carboxylic acid binding at the N_1_ position was not observed either. Single-point hydrogen bonding by carboxylic acids to pyridine nitrogen atoms can be found in the Cambridge Structural Database (CSD) [24]. However, the vast majority of those reported co-crystals are formed in the presence of aromatic carboxylic acids. In comparison there are a handful reported with aliphatic carboxylic acids [25,26]. Several of these have additional structure-directing interactions [27,28,29], and it is conceivable that these interactions facilitate intermolecularly driven assembly.

**Figure 9:**
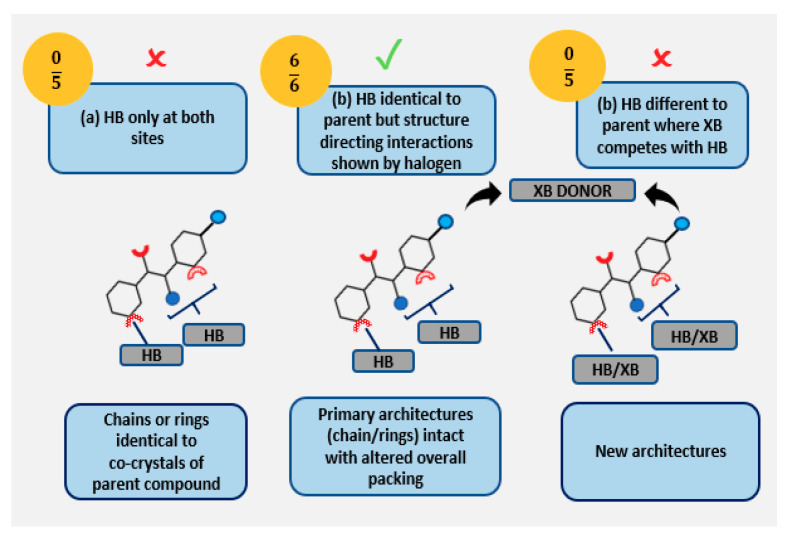
Schematic representation of effect of halogen on aggregation of molecules with halogens in crystal structures of 3Pyr-X.

All six co-crystals from the 3Pyr-X series showed identical hydrogen bonding as compared to those of the co-crystals of the same co-formers with the unhalogenated target. Thus, the combination of 3Pyr-X and odd-chain acids led to hydrogen-bonded chains, and 3Pyr-X and even-chain acids gave hydrogen-bonded rings. However, the halogen atom (bromine and iodine) showed structure directing interactions of the type X···O(OH) linking the hydrogen-bonded motifs together (Figure 9). The van der Waals reduction was 10% with the iodinated compounds and 5% in the brominated co-crystals, which is consistent with what is expected from the relative magnitudes of their corresponding sigma-holes [21].

**Figure 10:**
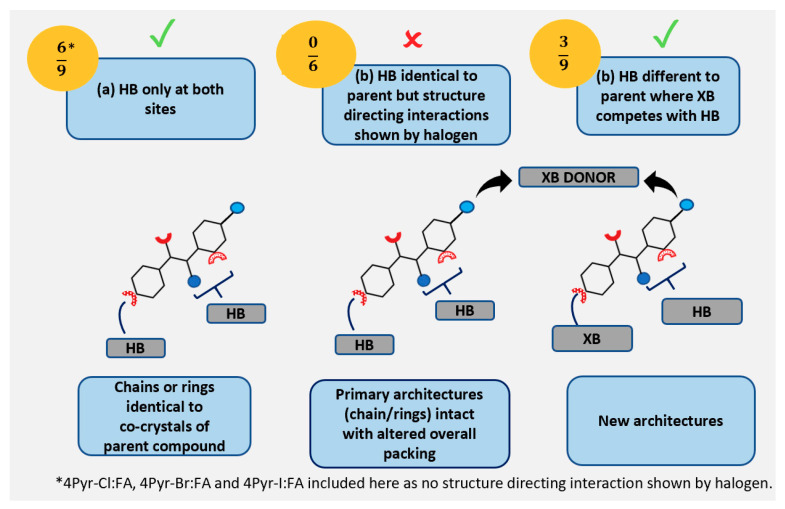
Schematic representation of effect of halogen on aggregation of molecules with halogens in crystal structures of 4Pyr-X.

In the co-crystals of the 4Pyr series halogenated compounds, all except 4Pyr-I:AA, 4Pyr-I:SuA, and 4Pyr-I:SeA showed hydrogen bonding at both the N_2_(py) and N_1_(py)-NH sites. In the three co-crystals of fumaric acid, both pockets were occupied by hydrogen bonding. However, –NH···O=C hydrogen bond interactions were observed instead of –COOH···N_1_(py)-NH seen in other co-crystals. Although, Type I short X···X contacts in 4Pyr-Br:FA and 4Pyr-I:FA were observed, these two co-crystals were isostructural to 4PyrCl:FA, and therefore these short contacts are more likely to be the result of simple close-packing instead of due to any structure directing interactions (Figure 10). The 4Pyr-I target showed hydrogen bonding only at the ···N_1_(py)-NH pocket, whilst the iodine atom formed an I···N_2_ near-linear halogen bond. This is the only instance where we saw the halogen bond competing with hydrogen bonding. The 4Pyr-I target was also the only compound in which the iodine atom exhibited competitive halogen bonding (with respect to hydrogen bonding) in the individual homomeric structures.

**Figure 11:**
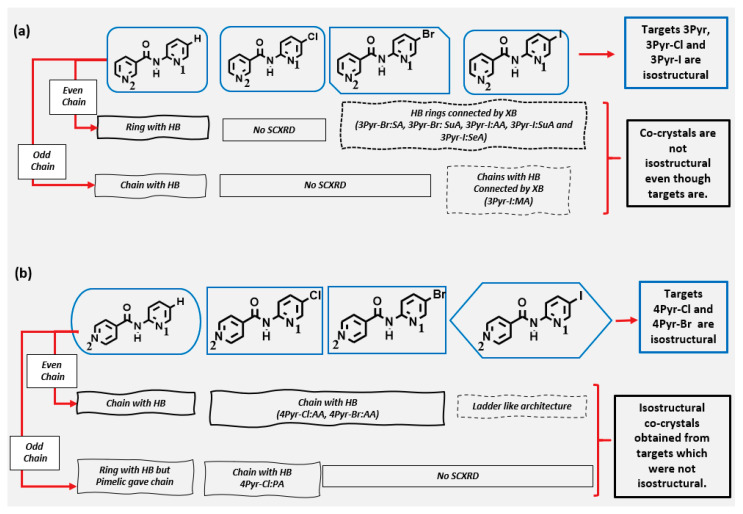
Map of isostructurality between targets and co-crystals of (**a**) 3Pyr and (**b**) 4Pyr series compounds. The shape of the box around targets indicate if they are isostructural or not.

The torsion angle between the two aromatic rings in the crystal structures of the individual 3Pyr-X target molecules ranged from ~30° to 50°, which is similar to the range found in the structures of the 4Pyr-X analogues, ~30° to 60° [21]. However, in the co-crystals of 3Pyr series, the target molecules were near planar, with torsion angles ranging from ~0° to 10°, whereas they fell in the ~10° to 45° range for the target molecules of the 4Pyr co-crystals. There is no obvious reason as to why the two groups behaved differently, but since the torsion in these targets followed a relatively shallow potential energy curve, crystal packing would clearly influence the molecular geometry in each group. It is notable that even though the individual targets 3Pyr and 3Pyr-I were isostructural, the analogues co-crystals were not, due to the I···O(OH) halogen bond. In contrast, although 3Pyr-Br and 3Pyr-I were not isostructural, the co-crystals formed by these two targets were. In the 4Pyr series, although the co-crystal formed by the three targets (4Pyr, 4Pyr-Cl, and 4Pyr-Br) were isostructural, these two halogenated targets were not isostructural with the corresponding parent. The co-crystal formed with the odd-chain pimelic acid and 4Pyr-Cl led to a chain rather than expected ring architecture, analogues to 4Pyr. Therefore, we can conclude that isostructurality is not directly transferrable from single component to multi-component systems (Figure 11).

## 4. Experimental

The target compounds were prepared as previously reported [21]. Co-crystal screening with ten aliphatic dicarboxylic acids (Figure 1) was carried out using liquid assisted grinding with stoichiometric 1:1 amounts of target and co-former, followed by IR characterization of the ground powder. A total of 100 experiments were performed. Broad stretches near 1850 cm^−1^ and 2450 cm^−1^ indicative of intermolecular O-H···N hydrogen bonds were used for confirming the presence of a co-crystal [20]. IR spectra of cocrystal screening experiments were recorded with a Nicolet 380 FT-IR spectrometer using an attenuated total reflection (ATR) technique and ZnSe as the crystal. Melting points were measured using a Fisher-Johns melting point apparatus or a TA Instruments DSC Q20 differential scanning calorimeter. Datasets for single-crystal X-ray diffraction analysis were collected on a Bruker Kappa APEX II system using CuKα radiation.

## 5. Conclusions

No co-crystals were obtained with the 2Pyr series since the omnipresent intramolecular hydrogen bonding prevented any opportunities for co-crystal synthesis via intermolecular interactions with any co-former. In the structures obtained from the 3Pyr and 4Pyr series where halogen atoms were engaged in halogen bonding (XB), the extent of these interactions, as indicated by reduction in combined van der Waals radii, was in the order of Cl < Br < I. Only 4Pyr-I exhibited halogen bonding in the homomeric state, as well as in three of four co-crystals. In the other six co-crystals of the 4Pyr series, no structure directing effects were shown by the halogen atoms. In the six co-crystals of the 3Pyr series, all showed complementary interactions to hydrogen bonding. Thus, in the fifteen co-crystals obtained from 3Pyr-X and 4Pyr-X targets, structure directing influence shown by the halogen was either none (6/15), complementary (6/15), or competitive (3/15) to hydrogen bonding. Since isostructural targets did not always lead to isostructural co-crystals, and since isostructural co-crystals were obtained with targets that were not isostructural, it is clear that isostructurality is not necessarily transferrable from single- to multi-component systems.

## Figures and Tables

**Table 1:** Results of Co-Crystal Screening Experiments. (**🗴** = negative, **✓** = positive, * = single crystal structure obtained).

	Malonic Acid	Glutaric Acid	Pimelic Acid	Azelaic Acid	Fumaric Acid	Succinic Acid	Adipic Acid	Suberic Acid	Sebacic Acid	Dodecanedioic Acid
**2Pyr**	**🗴**	**🗴**	**🗴**	**🗴**	**🗴**	**🗴**	**🗴**	**🗴**	**🗴**	**🗴**
**2Pyr-Cl**	**🗴**	**🗴**	**🗴**	**🗴**	**🗴**	**🗴**	**🗴**	**🗴**	**🗴**	**🗴**
**2Pyr-Br**	**🗴**	**🗴**	**🗴**	**🗴**	**🗴**	**🗴**	**🗴**	**🗴**	**🗴**	**🗴**
**2Pyr-I**	**🗴**	**🗴**	**🗴**	**🗴**	**🗴**	**🗴**	**🗴**	**🗴**	**🗴**	**🗴**
**3Pyr-Cl**	**✓**	**🗴**	**✓**	**🗴**	**🗴**	**✓**	**✓**	**✓**	**🗴**	**🗴**
**3Pyr-Br**	**✓**	**✓**	**🗴**	**🗴**	**✓**	✓*	**✓**	✓*	**🗴**	**🗴**
**3Pyr-I**	✓*	**✓**	**🗴**	**🗴**	**✓**	**✓**	✓*	✓*	✓*	**🗴**
**4Pyr-Cl**	**✓**	**✓**	✓*	**✓**	✓*	**✓**	✓*	**✓**	**✓**	**✓**
**4Pyr-Br**	**✓**	**✓**	**✓**	**✓**	✓*	**✓**	✓*	**✓**	**✓**	**✓**
**4Pyr-I**	**✓**	**✓**	**✓**	**✓**	✓*	✓*	✓*	**✓**	✓*	**🗴**

**Table 2:** Solvents used for Crystal Growth and Descriptions of Crystals.

Co-Crystal	Code	Solvent	Melting Point	Color and Morphology
(*N*-(5-bromopyridin-2-yl)nicotinamide) Succinic acid (1:1)	3Pyr-Br:SA	Ethyl acetate: MeOH(1:1)	185–187 °C	Colorless, Parallelepiped
(*N*-(5-bromopyridin-2-yl)nicotinamide) Suberic acid (1:1)	3Pyr-Br:SuA	Ethyl acetate: MeOH(1:1)	149–151 °C	Colorless, Rectangular
(*N*-(5-iodopyridin-2-yl)nicotinamide) Adipic acid (1:1)	3Pyr-I:AA	Ethyl acetate	163–165 °C	Yellow, Rectangular
(*N*-(5-iodopyridin-2-yl)nicotinamide) Suberic acid (1:1)	3Pyr-I:SuA	Ethyl acetate	148–150 °C	Colorless, Rectangular
(*N*-(5-iodopyridin-2-yl)nicotinamide) Sebacic acid (1:1)	3Pyr-I:SeA	Ethyl acetate	139–141 °C	Yellow, Rectangular
(*N*-(5-iodopyridin-2-yl)nicotinamide) Malonic acid (1:1)	3Pyr-I:MA	Chloroform: MeOH(1:1)	169–171 °C	Colorless, Rectangular
(*N*-(5-chloropyridin-2-yl)isonicotinamide) Adipic acid (1:1)	4Pyr-Cl:AA	Ethyl acetate	169–171 °C	Colorless, Rectangular
(*N*-(5-bromopyridin-2-yl)isonicotinamide) Adipic acid (1:1)	4Pyr-Br:AA	Acetonitrile	161–163 °C	Colorless, Rectangular
(*N*-(5-chloropyridin-2-yl)isonicotinamide) Pimelic acid (1:1)	4Pyr-Cl:PA	Ethyl acetate	163–165 °C	Colorless, Rectangular
(*N*-(5-iodopyridin-2-yl)isonicotinamide) Adipic acid (2:1)	4Pyr-I:AA	Acetonitrile	159–161 °C	Colorless, Chunk
(*N*-(5-iodopyridin-2-yl)isonicotinamide) Suberic acid (2:1)	4Pyr-I:SuA	Ethyl acetate: MeOH(1:1)	146–148 °C	Colorless, Rectangular
(*N*-(5-iodopyridin-2-yl)isonicotinamide) Subacic acid (2:1)	4Pyr-I:SeA	Ethyl acetate	147–149 °C	Colorless, Rhombus
(*N*-(5-chloropyridin-2-yl)isonicotinamide) Fumaric acid (1:1)	4Pyr-Cl:FA	Ethyl acetate	231–233 °C	Colorless, Irregular
(*N*-(5-bromopyridin-2-yl)isonicotinamide) Fumaric acid (1:1)	4Pyr-Br:FA	Methanol	240–242 °C	Colorless, Chunk
(*N*-(5-iodopyridin-2-yl)isonicotinamide) Fumaric acid (1:1)	4Pyr-I:FA	Ethyl acetate	237–239 °C	Colorless, Blocks

**Table 3:** Hydrogen- and Halogen-Bond Parameters in the Six 3Pyr-X Co-Crystals.

	D-H/X··A	D/X···A (Å)	D-H/X···A (deg)
3Pyr-Br:SA	N8-H8···O17	3.071(3)	167.(3)
	O18-H18···N2	2.732(3)	165.(5)
	O24-H24···N13	2.691(3)	172.(4)
	C4-Br7···O18	3.191(2)	158.56(8)
3Pyr-Br:SuA	N8-H8···O17	2.938(3)	166.(3)
	O18-H18···N2	2.692(3)	175.(3)
	O28-H28···N13	2.754(3)	177.(4)
	C4-Br7···O18	3.1979(19)	159.79(8)
3Pyr-I:AA	N8-H8···O17	3.198(4)	173.(4)
	O18-H18···N2	2.668(4)	171.(9)
	O26-H26···N13	2.683(4)	169.(7)
	C4-I7···O18	3.186(3)	155.15(11)
3Pyr-I:SuA	N8-H8···O27	2.970(5)	170.(4
	O18-H18···N13	2.766(6)	167.(7)
	O28-H28···N2	2.679(5)	167.(5)
	C4-I7···O28	2.086(4)	159.81(14)
3Pyr-I:SeA	O4B-H4B···N1D	2.692(5)	169.5
	N7D-H7D···O5B	3.088(6)	162.4(3)
	O2D-H2D ···N11D	2.718(6)	172.6
	C3D-I1D···O4D	3.227(4)	151.4(2)
	O2B-H2B···N11B	2.704(6)	165.0
	O4D-H4D···N1B	2.660(5)	171.2
	N7B-H7B···O5D	3.043(5)	170.1(3)
	C3B-I1B···O4B	3.229(4)	152.5(2)
	O4A-H4A···N1C	2.673(5)	171.3
	N7C-H7C···O5A	3.043(6)	168.0(3)
	O4C- H4C ···N11C	2.706(6)	172.2
	C3C-I1C···O2C	3.267(4)	150.9(2)
	O2A-H2A···N11A	2.705(6)	165.2
	O2C-H2C···N1A	2.685(5)	168
	N7A-H7A···O3C	3.068(6)	169.2(3)
	C3A-I1A···O4A	3.298(4)	151.1(2)
	04F-H4F···N1E	2.661(6)	169.4
	N7E-H7E···05F	3.050(6)	161.8(3)
	O18- H18 ···N1F	2.714(6)	169.1
	C3E-I1E···O4E	3.201(4)	152.4(2)
	O4E-H4E···N1F	2.665(5)	170.5
	N7F-H7F···O5E	3.068(5)	162.4(3)
	O23-H23··· N11F	2.661(6)	169.4
	C16-I1F···O4F	3.229(4)	152.5(2)
3Pyr-I:MA	N8-H8···O22	2.979(5)	170.(5)
	O18-H18···N13	2.634(5)	165.(6)
	O23-H23···N2	2.689(5)	173.(8)
	C4-I7···O17	3.231(4)	156.63(14)
	C4-I7···O23	3.452(3)	150.84(13)

**Table 4:** Hydrogen- and Halogen-Bond Parameters in the Nine 4Pyr-X Co-Crystals.

	D-H/I···A	D/I···A (Å)	D-H···A (deg)
4Pyr-Cl:AA	N8-H8···O25	2.7376(19	176(2)
	O18-H18···N14	2.678(2)	166(3)
	O26-H26···N2	2.7376(19)	172(3)
4Pyr-Br:AA	N8-H8···O17	2.942(2)	172(3)
	O18-H18···N2	2.735(2)	172(4)
	O26-H26···N14	2.683(3)	172(4)
4Pyr-I:AA	N8-H8···O33	2.828(4)	175.(5)
	N24-H24···O38	2.874(4)	167.(5)
	O34-H34···N2	2.689(4)	170.(6)
	O39-H39··· N18	2.718(4)	180.(10)
	C4-I7···N14	2.910(3)	173.99(10)
	C20-I23···N30	2.938(4)	177.38(13)
4Pyr-I:SuA	N8-H8···O33	2.877(6)	173.(5)
	N24-H24···O43	2.814(6)	167.(6)
	O34-H34···N2	2.765(6)	164.(16)
	O44-H44···N18	2.738(6)	172.(7)
	C4-I7···N14	3.140(5)	168.28(19)
	C4-I23···N30	2.931(5)	170.09(18)
4Pyr-I:SeA	N8-H8···O17	3.104(3)	166.(4)
	O18-H18···N2	2.697(3)	160.(6)
	C4-I7···N14	2.910(3)	173.99(10)
4Pyr-Cl:PA	N8-H8···O26	2.973(3)	174(3)
	O18-H18···N14	2.705(3)	174(4)
	O27-H27···N2	2.737(3)	169(3)
4Pyr-Cl:FA	N8-H8···O17	3.0940(15)	175.2(18)
	O18-H18···N14	2.6129(16)	168(2)
4Pyr-Br:FA	N8-H8···O17	3.094(2)	176.(3)
	O18-H18···N14	2.619(2)	162.1
	C4-Br7··· Br7	3.6187(5)	155.69(7)
4Pyr-I:FA	N8-H8···O17	3.118(4)	175.(3)
	O18-H18···N14	2.653(3)	167.(4)
	C4-I7··· I7	3.6478(7)	157.54(8)

## Data Availability

Crystallographic data (CCDC 2059879-2059892, 2059914 and 2059915) can be obtained free of charge via www.ccdc.cam.ac.uk/data_request/cif (accessed on 21 February 2021), or by emailing data_request@ccdc.cam.ac.uk, or by contacting The Cambridge Crystallographic Data Centre, 12 Union Road, Cambridge CB2 1EZ, UK; fax: +44 1223 336033.

## Supplementary Materials

The following are available online. Table S1: IR data of co-crystal screen, Table S2: Crystallographic data, Figure S3: 3Pyr-I:SeA with all crystallographically unique molecules displayed, Figure S4: 4Pyr-I:AA with all crystallographically unique molecules displayed.

## Sample Availability

Samples of the compounds are not available from the authors.
