# Supplementary material for: The Impact of Halogen Substituents on the Synthesis and Structure of Co-Crystals of Pyridine Amides
Source: Molecules. 2021 Feb 21;26(4):1147. doi: 10.3390/molecules26041147 (PMC7924845; doi:10.3390/molecules26041147)
Supplement: Supplementary file 1 [file molecules-26-01147-s001.pdf]

molecules

MDPI

Supporting Information

The Impact of Halogen Substituents on the Synthesis and Struc-Ture of Co-crystals of Pyridine Amides

Amila M. Abeysekera, Abhijeet S. Sinha and Christer B. Aakeröy*

[1]Department of Chemistry, Kansas State University, Manhattan, Kansas 66506
*+1-785-532-6096; Email:aakeroy@ksu.edu

S1. IR Data of Co-crystal Screen.
S2. Crystallographic Data.
S3. Figure of 3Pyr-I: SeA with all Crystallographically Unique Molecules Displayed.
S4. Figure of 4Pyr-I: AA with all Crystallographically Unique Molecules Displayed.

S1. IR Data of Co-crystal Screen.

		**Malonic Acid**	**Glutaric Acid**	**Pimelic Acid**	**Azelaic Acid**	**Fumaric Acid**	**Succinic Acid**	**Adipic Acid**	**Suberic Acid**	**Sebacic Acid**	**Dodecandioc Acid**
	c=o	1689	1677	1683	1686	1659	1679	1864	1685	1686	1684
		OH---N stretch wave number if present									
		C=O									
2Pyr	1686	x	x	x	x	x	x	x	x	x	x
2Pyr-Cl	1692	x	x	x	x	x	x	x	x	x	x
2Pyr-Br	1692	x	x	x	x	x	x	x	x	x	x
2Pyr-I	1689	x	x	x	x	x	x	x	x	x	x
3Pyr-Cl	1687	2489, 1919	x	2551, 1924	x	x	2486, 1942	2495, 1847	2507, 1944	x	x
		1716, 1694, 1680		1692			1701, 1680	1719, 1686	1683		
3Pyr-Br	1687	2440, 1883	2561, 1940	x	x	2459, 1872	2481, 1897	2538, 1868	2517, 1879	x	x
		1715, 1679	1682			1681	1700, 1682	1717, 1686	1683		
3Pyr-I	1669	2438, 1895	2454, 1895	x	x	2444, 1866	2484, 1889	2484, 1913	2,331	2516, 1901	x
		1678	1676			1677	1684, 1699	1695, 1683	1692, 1662	1685	
4Pyr-Cl	1676	2452, 1889	2457, 1934	2505, 1913	2499, 1896	2473, 1870	2480, 1905	2519, 1894	2504, 1911	2522, 1904	2456, 1885
		1753, 1680	1679	1697, 1681	1702, 1689, 1674	1665	1681	1689	1688	1692	1690
4Pyr-Br	1680	2470, 1892	2472, 1298	2503, 1904	2499, 1896	2443, 1887	2384, 1896	2457, 1861	2452, 1893	2528, 1905	2411, 1900
		1756, 1678	1698, 1680	1687	1703, 1675	1673	1670	1689	1687	1701, 1682	1690
4Pyr-I	1658	2489, 1897	2495, 1902	2516, 1942	2495, 1890	2394, 1888	2412, 1889	2438, 1933	2512, 1892	2551, 1904	x
		1686	1698, 1678	1696, 1687	1704, 1674	1673	1686, 1670	1686, 1676	1688	1698, 1674	

S2. Crystallographic Data.

**Code**	**3Pyr-Br:SA**	**3Pyr-Br:SuA**	**3Pyr-I:AA**	**3Pyr-I:SuA**	**3Pyr-I:SeA**	**3Pyr-I:MA**
Formula moiety	$(C_{11}H_8BrN_3O)$ $(C_4H_6O_4)$	$(C_{11}H_8BrN_3O)$ $(C_8H_{14}O_4)$	$(C_{11}H_8IN_3O)$ $(C_6H_{10}O_4)$	$(C_{11}H_8IN_3O)$ $(C_8H_{14}O_4)$	$(C_{11}H_8IN_3O)$ $(C_{10}H_{18}O_4)$	$(C_{11}H_8IN_3O)$ $(C_3H_4O_4)$
Empirical formula	$C_{15}H_{14}BrN_3O_5$	$C_{19}H_{22}BrN_3O_5$	$C_{17}H_{18}IN_3O_5$	$C_{19}H_{22}IN_3O_5$	$C_{21}H_{26}IN_3O_5$	$C_{14}H_{12}IN_3O_5$
Molecular weight	396.20	452.30	471.24	499.29	527.35	429.17
Color, Habit	Colorless, Parallelepiped'	Colorless, Rec-tangular	Yellow, Rectan-gular	Colorless, Rec-tangular	Yellow, Rec-tangular	Colorless, Rec-tangular
Crystal system	Triclinic	Monoclinic	Triclinic	Monoclinic	Monoclinic	Triclinic
Space group, *Z*	$P\bar{1}$, 2	P $2_1/n$, 4	$P\bar{1}$, 2	P $2_1/n$, 4	P $2_1/n$, 24	$P\bar{1}$, 2
a, Å	5.3367(5)	5.3877(2)	5.3703(2)	5.4009(2)	24.2049(13)	7.1156(4)
b, Å	12.7411(9)	24.8548(10)	12.8242(4)	25.0798(6)	26.1148(14)	9.4342(4)
c, Å	13.4815(11)	14.4670(6)	14.3232(5)	14.7541(5)	20.8819(12)	12.0753(6)
α, º	63.463(4)	90	109.6500(10)	90	90	107.873(4)
β, º	82.016(5)	95.381(2)	92.2770(10)	95.572(3)	93.759(2)	94.178(4)

γ, º	80.728(4)	90	101.2150(10)	90	90	97.669(3)
Volume, Å^3	807.06(12)	1928.74(13)	905.33(5)	1989.05(11)	13171.2(13)	759.01(7)
Density, g/cm^3	1.630	1.558	1.729	1.667	1.596	1.878
T, ºK	296.(2)	200.(2)	200.(2)	200.(2)	296.15	200.(2)
Crystal size, min × mid × max	0.030 × 0.065 × 0.280	0.035 × 0.080 × 0.270	0.050 × 0.100 × 0.190	0.020 × 0.045 × 0.050	0.05 × 0.07 × 0.25	0.020 × 0.020 × 0.075
X-ray wavelength, Å	1.54178	1.54178	1.54178	1.54178	1.54178	1.54178
μ, mm^{-1}	3.766	3.227	14.200	12.963	11.779	16.866
Trans min / max	0.42 / 0.90	0.48 / 0.90	0.17 / 0.54	0.56 / 0.78	0.3369/ 0.7531	0.36 / 0.73
θ_{min}, º	3.67	3.55	3.30	3.49	1.829	3.87
θ_{max}, º	69.98	68.20	70.23	70.43	68.386	69.94
Reflections						
collected	9705	11951	10929	16237	92910	15635
independent	2845	3432	3223	3674	23496	2682
observed	2599	2903	3137	2850	17875	2426
R_{int}	0.0453	0.0394	0.0393	0.0538	0.0726	0.0561
Threshold expression	$> 2\sigma(I)$	$> 2\sigma(I)$	$> 2\sigma(I)$	$> 2\sigma(I)$	$> 2\sigma(I)$	$> 2\sigma(I)$
No. parameters	229	265	247	265	1633	220
No. restraints	3	1	2	1	0	0
R_1 (observed)	0.0358	0.0335	0.0462	0.0388	0.0545	0.0343
wR_2 (all)	0.0993	0.0837	0.1113	0.1029	0.1559	0.0880
Goodness of fit (all)	1.066	1.073	1.100	1.073	1.012	1.046
ρ_{max}, ρ_{min}, e Å^{-3}	0.440, -0.589	0.282, -0.351	2.358, -1.871	0.705, -0.858	1.899, -1.190	1.276, -0.526
Completeness to 2θ limit	0.925	0.970	0.932	0.962	0.971	0.929

**Code**	**4Pyr-Cl:AA**	**4Pyr-Br:AA**	**4Pyr-I:AA**	**4Pyr-I:SuA**	**4Pyr-I:SeA**
Formula moiety	$(C_{11}H_8ClN_3O)$ $(C_6H_{10}O_4)$	$(C_{11}H_8BrN_3O)$ $(C_6H_{10}O_4)$	$2(C_{11}H_8IN_3O)$, $C_6H_{10}O_4$	$2(C_{11}H_8IN_3O)$ $(C_8H_{14}O_4)$	$C_{11}H_8IN_3O$, $0.5(C_{10}H_{18}O_4)$
Empirical formula	$C_{17}H_{18}ClN_3O_5$	$C_{17}H_{18}BrN_3O_5$	$C_{28}H_{26}I_2N_6O_6$	$C_{30}H_{30}I_2N_6O_6$	$C_{16}H_{17}IN_3O_3$
Molecular weight	379.79	424.25	796.35	824.40	426.22
Color, Habit	Colorless, Rectangular	Colorless, Rectangular	Colorless, Chunk	Colorless, Rectangular	Colorless, Rhombus
Crystal system	Monoclinic	Monoclinic	Triclinic	Monoclinic	Triclinic
Space group, *Z*	P 2_1/n, 4	P 2_1/n, 4	P$\bar{1}$, 2	P 2_1/n, 4	P$\bar{1}$, 2
a, Å	12.2032(5)	12.2529(3)	9.5127(4)	8.1076(4)	4.61210(10)
b, Å	20.1674(8)	20.3709(5)	11.9915(5)	15.8799(9)	13.3320(3)
c, Å	7.3645(3)	7.3786(2)	13.8647(6)	24.6728(13)	14.1286(3)
α, º	90	90	73.5940(10)	90	75.4830(10)
β, º	107.514(2)	107.2830(10)	87.7300(10)	98.0320(10)	81.5960(10)
γ, º	90	90	82.0360(10)	90	85.1590(10)
Volume, Å^3	1728.43(12)	1758.56(8)	1502.54(11)	3145.4(3)	830.94(3)
Density, g/cm^3	1.459	1.602	1.760	1.741	1.704
T, ºK	199.99	199.99	200.(2)	200.(2)	200.(2)
Crystal size, min × mid × max	0.02 × 0.045 × 0.37	0.11 × 0.13 × 0.21	0.055 × 0.075 × 0.165	0.067 × 0.200 × 0.240	0.020 × 0.045 × 0.045

X-ray wavelength, Å	1.54178	1.54178	1.54178	1.54178	1.54178
μ, mm^{-1}	2.272	3.498	16.878	16.148	15.303
Trans min / max	0.4499 / 0.7533	0.5386 / 0.7534	0.17 / 0.46	0.11 / 0.41	0.55 / 0.75
θ_{min}, °	3.798	3.778	3.32	3.32	3.26
θ_{max}, °	70.363	70.197	68.43	68.39	70.47
Reflections					
collected	12991	9525	13729	18656	17735
independent	3131	3125	5274	5557	2942
observed	2831	3081	4878	5185	2886
R_{int}	0.0886	0.0341	0.0369	0.0663	0.0392
Threshold expression	> $2\sigma(I)$	> $2\sigma(I)$	> $2\sigma(I)$	> $2\sigma(I)$	> $2\sigma(I)$
No. parameters	247	248	395	414	217
No. restraints	0	1	3	3	2
R_1 (observed)	0.0449	0.0384	0.0364	0.0585	0.0277
wR_2 (all)	0.1312	0.0935	0.0995	0.1572	0.0688
Goodness of fit (all)	1.071	1.150	1.111	1.041	1.094
ρ_{max}, ρ_{min}, e Å^{-3}	0.273, -0.392	1.173, -0.504	1.153, -1.525	1.302, -1.638	0.779, -0.707
Completeness to 2θ limit	0.947	0.938	0.953	0.960	0.922

**Code**	**4Pyr-Cl:PA**	**4Pyr-Cl:FA**	**4Pyr-Br:FA**	**4Pyr-I:FA**
Formula moiety	$C_{11}H_8ClN_3O$, C_7H_{12} O4	$C_{11}H_8ClN_3O$, $0.5(C_4H_4O_4)$	$C_{11}H_8BrN_3O$, $0.5(C_4H_4O_4)$	$C_{11}H_8IN_3O$, $0.5(C_4H_4O_4)$
Empirical formula	$C_{18}H_{20}ClN_3O_5$	$C_{13}H_{10}ClN_3O_3$	$C_{13}H_{10}BrN_3O_3$	$C_{13}H_{10}IN_3O_3$
Molecular weight	393.82	291.69	336.15	383.14
Color, Habit	Colorless, Rectangular	Colorless, Irregular	Colorless, Chunk	Colorless, Blocks
Crystal system	Monoclinic	Triclinic	Triclinic	Triclinic
Space group, Z	P $2_1/n$, 4	$P\bar{1}$, 2	$P\bar{1}$, 2	$P\bar{1}$, 2
a, Å	7.2579(4)	9.2874(5)	9.3345(9)	9.3944(9)
b, Å	21.6911(11)	9.3503(5)	9.3632(9)	9.4292(10)
c, Å	11.7229(8)	9.3983(5)	9.3854(9)	9.5060(9)
α, °	90	96.2480(10)	96.0860(10)	96.759(6)
β, °	90.298(4)	112.2120(10)	111.5860(10)	118.573(5)
γ, °	90	117.9830(10)	118.1120(10)	109.471(6)
Volume, Å^3	1845.53(19)	625.02(6)	632.54(11)	657.00(12)
Density, g/cm^3	1.417	1.550	1.765	1.937
T, °K	199.99	199.99	296.(2)	296.(2)
Crystal size, min × mid × max	0.035 × 0.06 × 0.1	0.06 × 0.08 × 0.34	0.090 × 0.140 × 0.190	0.084 × 0.284 × 0.518
X-ray wavelength, Å	1.54178	1.54178	1.54178	0.71073
μ, mm^{-1}	2.148	2.831	4.555	2.448
Trans min / max	0.6321 / 0.7533	0.5682 / 0.7530	0.48 / 0.69	0.36 / 0.82
θ_{min}, °	4.076	5.632	5.39	2.43
θ_{max}, °	70.056	67.991	67.72	25.81
Reflections				
collected	15240	5844	6061	16783
independent	3395	2183	2210	2508
observed	2662	2076	2181	2097
R_{int}	0.0478	0.0365	0.0293	0.0394
Threshold expression	> $2\sigma(I)$	> $2\sigma(I)$	> $2\sigma(I)$	> $2\sigma(I)$
No. parameters	256	189	187	189
No. restraints	2	0	0	1
R_1 (observed)	0.0490	0.0322	0.0293	0.0269

wR_2 (all)	0.1431	0.0885	0.0842	0.0566
Goodness of fit (all)	1.035	1.062	1.150	1.067
ρ_{max}, ρ_{min}, e Å^{-3}	0.327, -0.250	0.224, -0.230	0.367, -0.346	0.537, -0.624
Completeness to 2θ limit	0.969	0.958	0.963	0.987

S3. Figure of 3Pyr-I: SeA with all Crystallographically Unique Molecules Displayed.

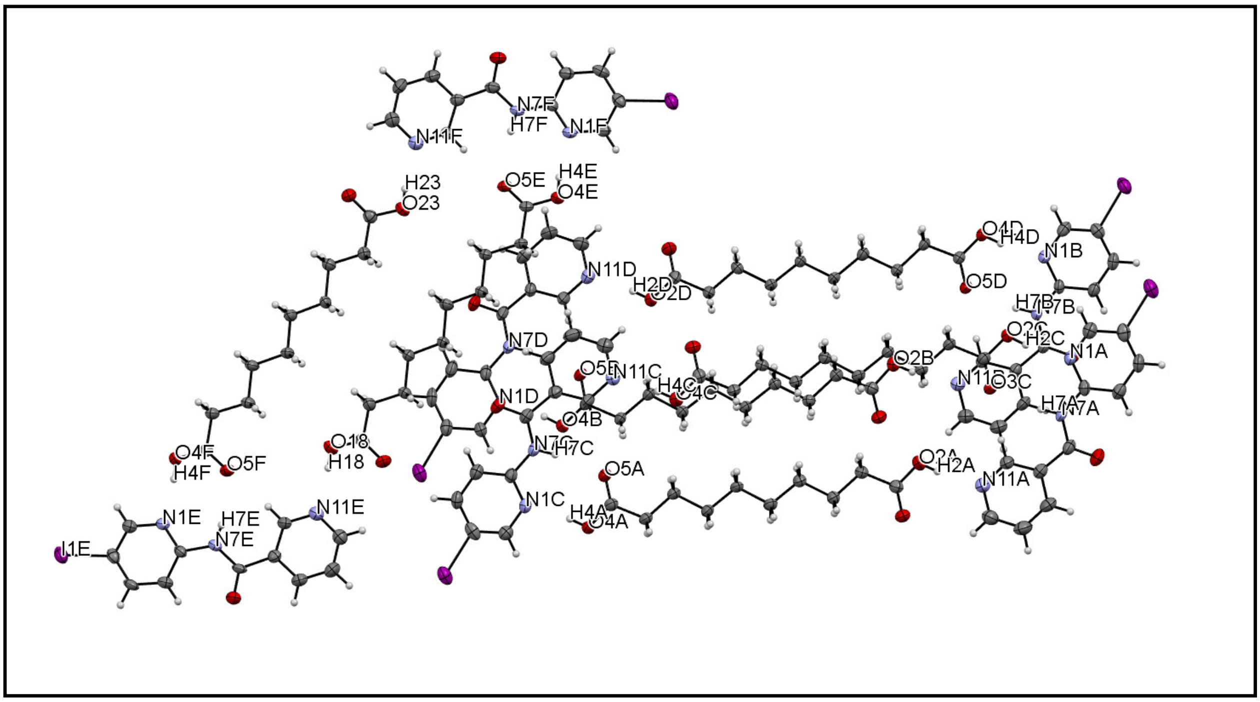

Figure 1. 3Pyr-I:SeA with all crystallographically unique molecules displayed.

S4. Figure of 4Pyr-I:AA with all Crystallographically Unique Molecules Displayed.

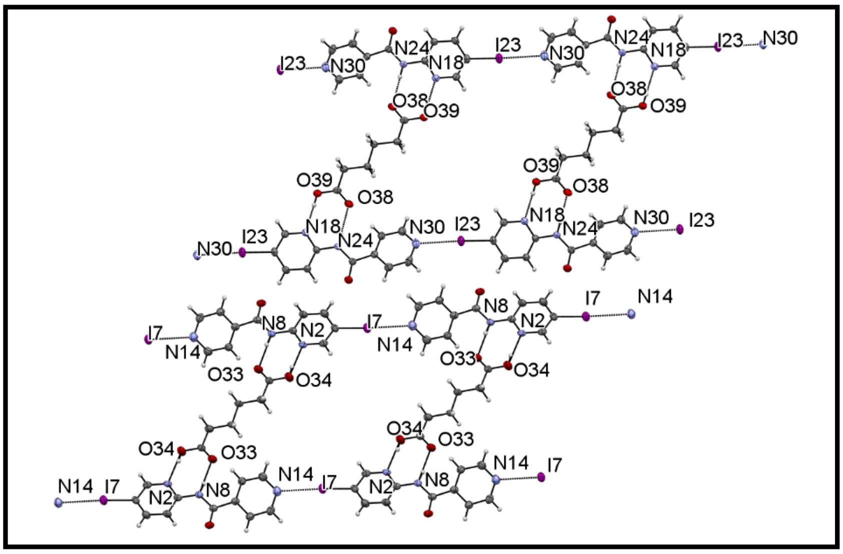

Figure 2. 4Pyr-I:AA with all crystallographically unique molecules displayed.
